# Detection of Human Papillomavirus DNA by AffiProbe HPV-DNA Test Kit in Cervical Scrapes or Biopsies-Histopathologic Correlates

**DOI:** 10.1155/S1064744994000517

**Published:** 1994

**Authors:** Pekka Nieminen, Tarja Jalava, Arja Kallio, Marjut Ranki, Jorma Paavonen

**Affiliations:** 1 Department of Obstetrics and Gynecology University Hospital Haartmaninkatu 2 Helsinki 00290 Finland; 2 Orion Corporation Orion Pharmaceutica/Biotechnology Helsinki Finland

## Abstract

*Objective:* The aim of this study was to evaluate and compare the efficacy of punch biopsies and cervical scrapes in the detection of human papillomavirus (HPV) DNA from the cervix and compare the results with the histopathologic diagnosis.

*Methods:* The specimens were collected simultaneously, and HPV DNA was detected using a liquid hybridization test.

*Results:* Biopsies and scrapes were equally efficient, but each detected only two-thirds of all HPV-DNA-positive patients. Thus, the positivity rate increased when both tests were used. Overall, 13% of patients with normal histopathology, 38% of patients with benign atypia, and 66% of patients with squamous intraepithelial lesions (SIL) were HPV-DNA positive. HPV-DNA 16 was found in 54% of HPV-DNA-positive patients with SIL, in 20% of HPV-DNA-positive patients with atypia, and in none of patients with normal histopathology.

*Conclusions:* The liquid hybridization test used in this study detects HPV DNA equally efficiently from both biopsies and scrapes. The test can be performed in 1 working day. However, the sensitivity of the test is low, and it only detects a limited number of HPV types.


Infectious Diseases in Obstetrics and Gynecology 2:126–129 (1994)
© 1994 Wiley-Liss, Inc.

### Detection of Human Papillomavirus DNA by AffiProbe HPV-DNA Test Kit in Cervical Scrapes or Biopsies-Histopathologic Correlates

**Pekka Nieminen, Tarja Jalava, Arja Kallio, Marjut Ranki, and Jorma Paavonen**

*Department of Obstetrics and Gynecology, University Hospital (P.N., J.P.), and Orion Corporation, Orion PharmaceuticalBiotechnology (T.J., A.K., M.R.), Helsinki, Finland*

### Abstract

***Objective:*** **The aim of this study was to evaluate and compare the efficacy of punch biopsies and cervical scrapes in the detection of human papillomavirus (HPV) DNA from the cervix and compare the results with the histopathologic diagnosis.**

***Methods:*** **The specimens were collected simultaneously, and HPV DNA was detected using a liquid hybridization test.**

***Results:*** **Biopsies and scrapes were equally efficient, but each detected only two-thirds of all HPV-DNA-positive patients. Thus, the positivity rate increased when both tests were used. Overall, 13% of patients with normal histopathology, 38% of patients with benign atypia, and 66% of patients with squamous intraepithelial lesions (SIL) were HPV-DNA positive. HPV-DNA 16 was found in 54% of HPV-DNA-positive patients with SIL, in 20% of HPV-DNA-positive patients with atypia, and in none of patients with normal histopathology.**

***Conclusions:*** **The liquid hybridization test used in this study detects HPV DNA equally efficiently from both biopsies and scrapes. The test can be performed in 1 working day. However, the sensitivity of the test is low, and it only detects a limited number of HPV types.** © 1994 Wiley-Liss, Inc.

Key words

HPV DNA, cervix, liquid hybridization, biopsy

Infection with specific types of human papillomavirus (HPV) is associated with different types of cervical lesions. Therefore, HPV typing has become increasingly important.[1–4] Many techniques are currently available for HPV typing.[3,5–7] Sandwich hybridization in liquid phase is a rapid typing technique with a comparable sensitivity and specificity to the more commonly used dot blot technique.[8–10] Cervical scrapes have been predominantly used in previous studies of commercially available HPV-DNA test kits.[3,8–11] Only a few studies have compared cervical scrapes and cervical biopsies in the detection of HPV DNA.[12,13] However, since patients with even mild degrees of cytologic atypia will be usually evaluated by colposcopy and directed biopsy,[14,15] it would be meaningful to also use the biopsy material for HPV typing.

In this study, we used a commercially available liquid hybridization test that allows detection and typing of HPV-DNA types 6/11, 18/33, 16, and 31. We compared the HPV-DNA typing results from cervical scrapes and cervical biopsies collected simultaneously from patients with cytologic atypia referred for colposcopy.

**Address correspondence/reprint requests to Dr. Pekka Nieminen, Department of Obstetrics and Gynecology, University Hospital, Haartmaninkatu 2, 00290 Helsinki, Finland.**

Received March 1, 1994

Accepted June 23, 1994

### SUBJECTS AND METHODS

The study population consisted of 202 patients with cytologic atypia referred for colposcopy. The reason for referral was dyskaryotic cytologic changes consistent with low- or high-grade squamous intraepithelial lesions (SIL) in 119 cases, atypical squamous cells of undetermined significance in 72 cases, glandular-cell atypia in 8 cases, and other inflammatory changes in 3 cases.

### Collection of Specimens

During each patient's speculum examination, a cellular-scrape specimen for HPV-DNA testing was collected using the AffiProbe HPV specimen collection kit (Orion Corporation, Orion Pharmaceutica, Helsinki). A colposcopy was next performed. The Olympus OCS colposcope (Olympus Optical Co. Ltd., Tokyo, Japan) or Zeiss colposcope (Zeiss AG, Germany) was used throughout the study. Standard terminology was used to describe colposcopic findings.[16] Colposcopically directed biopsies were obtained from the most representative areas of the atypical transformation zone of the cervix. Parallel sections of biopsy specimens obtained from each lesion were used for routine histopathologic examination and HPV-DNA testing.

The scrape specimens were processed during the same day. Cells were recovered from the sample medium after centrifugation for 5 min at 2,000 rpm. The cells in approximately 150 μl of the sample medium were frozen at −20°C until tested by the AffiProbe HPV-DNA test kit. The biopsy specimens were kept frozen in the sample medium of the AffiProbe biopsy collection kit until tested. Cervical biopsies were pretreated with proteinase K digestion overnight at 37°C to release the viral DNA.

### HPV-DNA Testing

The adequacy of the specimens was verified by the AffiProbe HPV-Specimen Adequacy test kit (Orion Corporation, Orion Pharmaceutica).[17] The AffiProbe HPV-DNA test kit allows simultaneous detection and typing of HPV-DNA 6/11, 18/33, 16, and 31. It is based on sandwich hybridization in solution followed by affinity-based hybrid collection (Orion Corporation, Orion Pharmaceutica).[10] The specimens were first divided in 4 30-μl aliquots, each of which was treated separately with specimen pretreatment solution to denature the viral DNA. The aliquots were next subjected to hybridization in solution for 3 h with type-specific DNA-detector probes labeled with $^{35}S$ isotope and biotinylated capture DNA probes. The formed hybrids were subsequently collected onto streptavidin-coated microtiter plates and washed, and the eluted detector probes were counted in a liquid scintillation counter. The cutoff of the test was set according to the mean of 2 low-positive standards included in the kit.[8,10] In all cases, the cutoff was at least 1.5 times the mean of the background. The specimens were considered positive if the ratio of the signal to the cutoff obtained was ≥1. Since the AffiProbe HPV-DNA test shows cross-reaction for HPV types 16 and 31 if more than $10^7$ molecules are present, the following rules, based on experiments with cloned HPV DNAs, were adapted to rule out cross-reactions. If the signal-to-cutoff ratio for 1 of the types was higher than 10 and for the other type between 1 and 3, the latter finding was considered the cross-reaction. If the signal-to-cutoff ratio was lower than 10 for both types, the specimen was considered positive for both types.

**TABLE 1. Comparison of cervical scrape and biopsy in the detection of HPV DNA by AffiProbe test**

| | Biopsy | | |
|---|---|---|---|
| Scrape | Positive | Negative | Total |
| Positive | 24 | 30 | 54 |
| Negative | 26 | 122 | 148 |
| Total | 50 | 152 | 202 |

### RESULTS

Satisfactory cervical scrapes and cervical biopsies were obtained from 202 patients, of whom 80 (40%) were positive for HPV-DNA 6/11, 18/33, 16, or 31 by cervical scrape or biopsy and 122 were negative for HPV DNA by both specimens.

Of the 80 HPV-DNA-positive patients, cervical scrape alone detected 54 (67.5%) and cervical biopsy alone detected 50 (62.5%) (Table 1). The performance of the cervical scrape or biopsy in the detection of specific HPV-DNA types is shown in Table 2. HPV-DNA 16 was found 2 times more often in cervical scrapes than in cervical biopsies. Other HPV-DNA types were found to be equally common in both types of specimens.

**TABLE 2. Number of positive specimens by HPV-DNA type**

| | HPV-DNA type | | | |
|---|---|---|---|---|
| Positive specimen | 6/11 | 18/33 | 16 | 31 |
| Scrape (N = 54)[a] | 10 | 19 | 25 | 12 |
| Biopsy (N = 50)[b] | 13 | 19 | 13 | 12 |

[a]Thirteen patients had >1 HPV-DNA types.
[b]Six patients had >1 HPV-DNA types.

**TABLE 3. Histopathologic findings in relation to HPV-DNA positivity from cervical scrape or biopsy**

| | HPV-DNA[a] positive | | |
|---|---|---|---|
| Histopathologic finding | Biopsy | Scrape | Biopsy or scrape |
| Normal (N = 21) | 2 | 2 | 3 (13%) |
| Atypia (N = 152) | 35 | 34 | 58 (38%) |
| SIL (N = 29) | 13 | 14 | 19 (66%) |

[a]HPV-DNA 6/11, 18/33, 16 or 31.

**TABLE 4. Correlation of histopathology and detection of specific HPV-DNA types in biopsies**

| | HPV-DNA type in biopsy | | | | |
|---|---|---|---|---|---|
| Histopathology | 6/11 | 18/33 | 16 | 31 | Mixed |
| Normal (N = 21) | 0 | 1 | 0 | 1 | 0 |
| Atypia (N = 152) | 10 | 12 | 5 | 3 | 5[a] |
| SIL (N = 29) | 1 | 2 | 6 | 3 | 1[b] |

[a]Two had HPV-DNA 6/11 and 18/33, 2 had HPV-DNA 16 and 31, and 1 had HPV-DNA 31 and 18/33.

[b]HPV-DNA 16, 31, and 18/33 positive.

Biopsy showed normal histopathology in 21 patients, mild atypia in 152 patients, and SIL in 29 patients. HPV DNA was detected in 3/21 (13%) patients with normal histopathology, in 58/152 (38%) patients with atypia, and in 19/29 (66%) patients with SIL (Table 3). As shown in Table 3, there were no differences in the detection of HPV DNA in the different categories of cervical lesions, or from normal cervix whether studied from cervical scrape or biopsy.

Table 4 shows the correction between the histopathologic findings and the detection of specific HPV-DNA types in a biopsy taken from the same lesion. HPV-DNA 16 was detected in 7/13 (54%) HPV-DNA-positive biopsies in patients with SIL, in 7/35 (20%) HPV-DNA-positive patients with atypia, and in none of the biopsies taken from HPV-DNA-positive patients with normal histopathology. HPV-DNA 6/11 was detected in only 1 (8%) patient with SIL and in 12 (34%) patients with atypia. Two or more HPV-DNA types were detected in 1 (8%) patient with SIL and in 5 (14%) patients with atypia.

### DISCUSSION

The AffiProbe liquid hybridization test used in this study is suitable for screening large numbers of specimens. The test can be performed in 1 working day. In a previous study, we evaluated the same test kit in the detection and typing of HPV DNAs from cervical and vaginal scrapes.[8,9] We found that the sensitivity was comparable to that of a commercially available dot blot test (ViraType, Digene Diagnostics, Beltsville, MD).

In this study, we compared the detection and typing of HPV DNA from cervical scrapes and colposcopically directed biopsies from patients with normal cervix, patients with benign atypia, and patients with SIL. Our goal was to find out whether biopsy could also be used for HPV-DNA detection by AffiProbe.

We found that either one of the sampling procedures gave two-thirds of all positive results obtained by combining both sampling procedures. Although a cervical scrape most likely represents a larger epithelial area than a biopsy, the overall positivity rate by scrape alone or biopsy alone was almost identical. It was not surprising that a biopsy was not better than a scrape since a biopsy represents only a small area of the lesion and probably a smaller amount of viral DNA.

We found that combining scrape and biopsy increased the overall positivity rate of all HPV-DNA types. Likewise, a combined scrape collected from several genital epithelial surfaces is more representative than any single scrape from 1 location only. On the other hand, cervical scrape detects 66% of all HPV-DNA 16-positive specimens, whereas biopsy detects only 34%, suggesting that HPV-DNA 16 infection is diffuse, affecting large areas of the cervicovaginal mucosa.

The findings from the present study indicate that performing both scrapes and biopsies would increase the sensitivity. However, it would also add to the cost and therefore cannot be recommended. Since the scrape was more sensitive than the biopsy in the detection of HPV-DNA 16, switching to a biopsy specimen for HPV testing cannot be recommended either.

As shown in many previous studies,[1,4,5] there was an increase in the overall HPV-DNA positivity with increasing severity of the lesion. Furthermore, the rate of HPV-DNA 16 positivity increased from 0 in normal cervix to 54% in SIL when both HPV-DNA testing and histopathologic examination were performed on biopsies obtained from the same lesion.

One obvious problem was that the overall sensitivity of the test appeared to be quite low. It is somewhat disturbing that fewer than half the patients with any atypia and only two-thirds of the patients with histologically confirmed SIL had HPV DNA detected by either specimen.

### REFERENCES

1. Lorincz AT, Reid R, Jenson B, Greenberg MD, Lancaster W, Kurman RJ: Human papillomavirus infection of the cervix: Relative risk associations of 15 common anogenital types. Obstet Gynecol 79:328–337, 1992.
2. Cuzick J, Terry G, Ho L, Hollingworth T, Anderson M: Human papillomavirus type 16 DNA in cervical smears as predictor of high-grade cervical cancer. Lancet 339:959–960, 1992.
3. Roman A, Fife KH: Human papillomaviruses. Are we ready to type? Clin Microbiol Rev 2:166–190, 1989.
4. Schiffman MH: Recent progress in defining the epidemiology of human papillomavirus infection and cervical neoplasia. J Natl Cancer Inst 84:394–398, 1992.
5. Paavonen J, Koutsky LA, Kiviat N: Cervical neoplasia and other STD related genital and anal neoplasias. In Holmes KK, et al. (eds): Sexually Transmitted Diseases. 2nd ed. New York: McGraw-Hill, pp 561–592, 1990.
6. de Villiers E-M: Hybridization methods other than PCR: An update. In Munoz N, Bosch FX, Shah KV, Meheus A (eds): The Epidemiology of Human Papillomavirus and Cervical Cancer. IARC Scientific Publications No. 119. International Agency for Research on Cancer (WHO, Lyon, France), pp 111–119, 1992.
7. Gravitt PE, Manos MM: Polymerase chain reaction-based methods for the detection of human papillomavirus DNA. In Munoz N, Bosch FX, Shah KV, Meheus A (eds): The Epidemiology of Human Papillomavirus and Cervical Cancer. IARC Scientific Publications No. 119. International Agency for Research on Cancer (WHO), pp 121–134, 1992.
8. Ranki M, Leinonen AW, Jalava T, Nieminen P, Soares VRX, Paavonen J, Kallio A: Use of Affiprobe HPV test kit for detection of human papillomavirus DNA in genital scrapes. J Clin Microbiol 28:2076–2081, 1990.
9. Scandinavian Multicenter Study Group: Polymerase chain reaction and direct DNA tests in detection of human papillomavirus (HPV) DNA in cytologically normal and abnormal cervical smears. Acta Obstet Gynecol Scand 71:98–103, 1992.
10. Jalava T, Kallio A, Leinonen AW, Ranki M: A rapid solution hybridization method for detection of human papillomaviruses. Mol Cell Probes 4:341–352, 1990.
11. Nieminen P, Soares VRX, Aho M, Vesterinen E, Vaheri A, Paavonen J: Cervical human papillomavirus deoxyribonucleic acid and cytologic evaluations in gynecologic outpatients. Am J Obstet Gynecol 164:1265–1269, 1991.
12. Gitsch G, Reinthaller A, Tatra G, Breitenecker G: Diagnosis of cervical intraepithelial neoplasia and human papillomavirus infection: Punch biopsy versus cervical smear. Arch Gynecol Obstet 249:179–184, 1991.
13. Ask E, Jenkins A, Kaern J, Trope C, Kristiansen BE: Comparison of HPV detection in parallel biopsies and cervical scrapes by PCR. APMIS (Acta Pathologica et Microbiologica Scandinavica) 100:752–756, 1992.
14. Bigrigg MA, Codling BW, Pearson P, Read MD, Swingler GR: Colposcopic diagnosis and treatment of cervical dysplasia at a single clinic visit. Br Med J 336:229–231, 1990.
15. Paavonen J, Kiviat N, Wölner-Hanssen P, Stevens CE, Vontver L, Critchlow C, DeRouen T, Holmes KK: Natural history of cervical cytologic atypia in a sexually transmitted disease clinic population. Acta Cytol 33:831–838, 1989.
16. Paavonen J, Kiviat N, Wölner-Hanssen P, Stevens CE, Critchlow C, DeRouen T, Koutsky L, Holmes KK: Colposcopic manifestations of cervical and vaginal infections. Obstet Gynecol Surv 43:373–381, 1988.
17. Mäenpää J, Arstila P, Ranki M: Human papillomavirus detection from the female genital tract: Combined vs. separate scrape methods. Eur J Obstet Gynecol Reprod Biol 44:209–213, 1992.
